# Crystal structure of 7,8,15,16,17-penta­thiadi­spiro­[5.2.5^9^.3^6^]hepta­deca­ne

**DOI:** 10.1107/S2056989019007138

**Published:** 2019-05-24

**Authors:** Robert Hofstetter, Benedict J. Elvers, Felix Potlitz, Andreas Link, Carola Schulzke

**Affiliations:** a Institut für Pharmazie, Universität Greifswald, Friedrich-Ludwig-Jahn-Strasse 17, 17489 Greifswald, Germany; b Institut für Biochemie, Universität Greifswald, Felix-Hausdorff-Strasse 4, 17489 Greifswald, Germany

**Keywords:** crystal structure, sulfur-rich heterocycles, spiro compounds, lenthio­nine derivative

## Abstract

The title compound, C_12_H_20_S_5_, crystallizes in the monoclinic space group *P*2_1_/c with four mol­ecules in the unit cell. In the crystal, the asymmetric unit comprises the entire mol­ecule with the three cyclic moieties arranged in a line. The mol­ecules in the unit cell pack in a parallel fashion, with their longitudinal axes arranged along a uniform direction. The packing is stabilized by the one-dimensional propagation of non-classical hydrogen-bonding contacts between the central sulfur atom of the S_3_ fragment and the C—H of a cyclo­hexyl group from a glide-related mol­ecule [C⋯S = 3.787 (2) Å].

## Abstract 2

A di­spiro compound bearing two cyclo­hexyl moieties and a central sulfur-rich seven-membered ring was re-synthesized and newly crystallized. The modified synthetic procedure, the compound’s purification and characterization and the crystal structure – which is only the second of its kind – including non-classical hydrogen-bonding inter­actions are reported and discussed.

## Chemical context   

Cyclic polysulfides comprise a subgroup of pharmacologically inter­esting organosulfur compounds that – depending on constitution and conformation – have been shown to exert specific anti­bacterial, anti­fungal, allelopathic and cytotoxic activity (Davison & Sperry, 2017 ▸), as well as a plethora of H_2_S-mediated effects relying on H_2_S formation (Szabo & Papapetropoulos, 2017 ▸). The benefits imparted by these compounds have led to the evolution of synthetic pathways in many natural products, as well as organoleptic detection mechanisms in organisms confronted by them, including the senses of smell and taste in humans. Thus, volatile organic polysulfanes are among the most odorous compounds in natural products, including meat (Zhao *et al.*, 2019 ▸), plants (Liang *et al.*, 2017 ▸), and algae (Block *et al.*, 2017 ▸). In fungi, 1,2,4-tri­thiol­ane, 1,2,4,6-tetra­thiepane, and 1,2,3,4,5,6-hexa­thiepane have been found to contribute to the unique aroma of shiitake (*Lentnius edodes*), but it is 1,2,3,5,6-penta­thiepane (*lenthio­nine*) that combines the most potent biological (anti­bacterial, anti­fungal, and anti-coagulative) and sensory activity (Davison & Sperry, 2017 ▸). Structural characterizations of this type of compounds are rather rare. The very few reports available in the literature include the crystal structures of penta­thiepanes featuring two vicinal carbon atoms (Sugihara *et al.*, 1999 ▸). The conformational study of the title compound, 7,8,15,16,17-penta­thiadi­spiro­[5.2.5^9^.3^6^]hepta­dec­ane (C_12_H_20_S_5_), is supposed to aid in the elucidation of the mechanism of action by which naturally occurring and synthetic penta­thiepanes exert potent activity (Behnisch-Cornwell *et al.*, 2019 ▸) and to advance the application of lenthio­nine derivatives for medical and material purposes (Tanagi *et al.*, 2019 ▸).
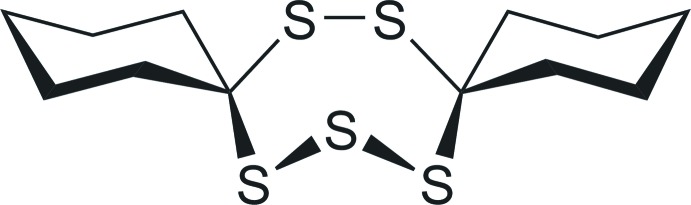


## Structural commentary   

**Figure 1:**
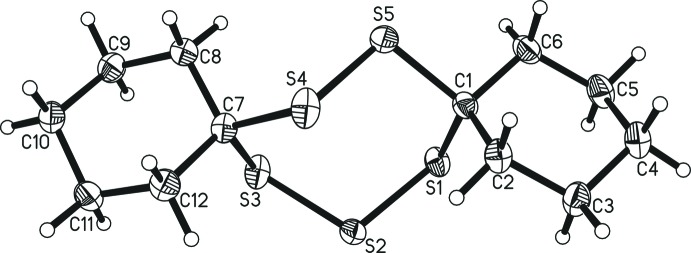
The mol­ecular structure of 7,8,15,16,17-penta­thiadi­spiro­[5.2.5^9^.3^6^]hepta­decane. Ellipsoids are shown at the 50% level.

**Figure 2:**
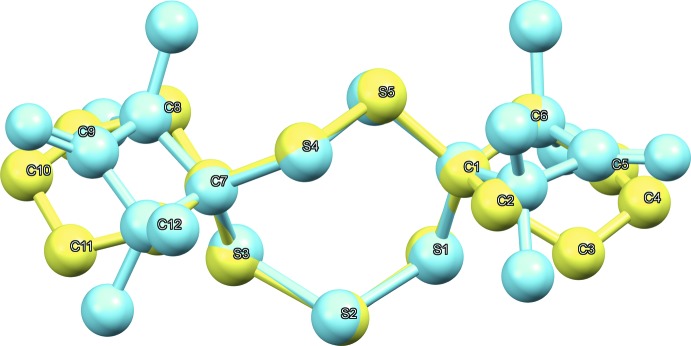
An overlay (*Mercury;* Macrae *et al.*, 2006 ▸) of 7,8,15,16,17-penta­thiadi­spiro­[5.2.5^9^.3^6^]hepta­decane (yellow) and the related structure from the CSD (blue, CSD refcode: MOSYOI; Mloston *et al.*, 2002 ▸). Only the atom labels for the title compound are shown; H atoms are omitted for clarity.

The title compound 7,8,15,16,17-penta­thiadi­spiro­[5.2.5^9^.3^6^]hepta­decane, C_12_H_20_S_5_, crystallizes in the monoclinic space group *P*2_1_/c. The mol­ecule constitutes the asymmetric unit while *Z* =4. The title compound consists of three rings in a corner-sharing juxtaposed arrangement (Fig. 1 ▸). The two outer cyclo­hexyl rings are both in a typical, rather unremarkable, chair conformation. They are connected to the central ring *via* spiro carbon atoms, which are tethered to each other by one S_2_ and one S_3_ moiety, thereby forming the central seven-membered ring. Crystal structures of such heterocyclic rings bearing five sulfur atoms in groups of two and three plus two carbon atoms are extremely rare, with only one example being available to date (Mloston *et al.*, 2002 ▸; refcode: MOSYOI in the CSD, version 5.40, March 2019; Groom *et al.*, 2016 ▸). In MOSYOI, the cyclo­hexyl substituents of this structure are replaced by 2,2,4,4-tetra­methyl­cyclo­butan-1-one moieties. The arrangement of the seven atoms of the central ring appears to be quite inflexible, at least in the solid state, as emphasized by an overlay of the two structures (Fig. 2 ▸), which shows distances of the overlayed atoms all well below 0.1 Å and an r.m.s. deviation of 0.0846 Å. Considering that the four-membered and heavily substituted rings in MOSYOI (Mloston *et al.*, 2002 ▸) are much more strained than the cyclo­hexane rings in the title compound, the conserved conformation of the central ring points towards the observed arrangement being thermodynamically rather favorable. In the context of investigating these compounds as pharmaceutical leads, such highly conserved structural motives are quite beneficial. The mean planes of the two cyclo­hexane rings, calculated from the positions of the six carbon atoms (*Mercury* software; Macrae *et al.*, 2006 ▸), enclose an angle of 21.96 (9)°, *i.e.* the two rings are not coplanar. The angles between the plane calculated from the positions of all seven atoms of the central ring and both cyclo­hexane-derived planes are 82.90 (5)° (C1 → C6) and 76.79 (5)° (C7 → C12), which are both close to perpendicular. This is similar in MOSYOI, with the four-membered rings being nearly perpendicular to the central seven-membered ring. The sulfur–sulfur bond distances range from 2.026 (1) to 2.035 (1) Å, which is a little bit shorter than the sum of the covalent radii of 2.06 Å (Pyykkö & Atsumi, 2009 ▸). The shortest S—S distance is between the two sulfur atoms of the S_2_ moiety, while the S_3_ moiety is slightly unsymmetrical [2.028 (1) and 2.035 (1) Å]. The shorter S—S bond in the S_3_ moiety is the one that points towards a non-classical hydrogen-bonding contact (*vide infra*), implying that this inter­action might influence the relative distances in the S_3_ fragment. The angles involving central S atoms range from 105.16 (5)° (around S1) to 106.89 (5)° (around S3) while the S—C—S angles are slightly wider with 111.58 (7)° (around C1) and 114.31 (7)° (around C7), *i.e*. they are more and less acute, respectively, than the ideal tetra­hedral angle.

## Supra­molecular features   

**Figure 3:**
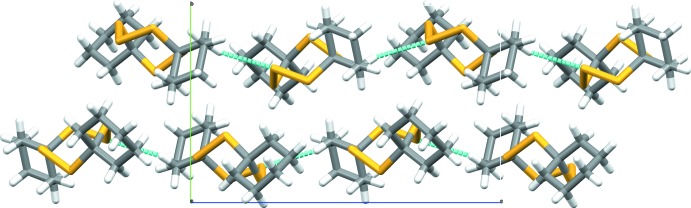
Crystal packing view along the *a* axis showing the non-classical hydrogen-bonding contacts (blue) protruding along the *c-*axis direction (analyzed and drawn with *Mercury;* Macrae *et al.*, 2006 ▸).

In the crystal packing all mol­ecules are oriented along parallel lines, although turned/flipped alternately by roughly 180° around the mol­ecules’ approximate longitudinal axes through the three rings which rest on crystallographic glides in the *ac* planes. The crystallographic direction of these vectors approximates [40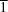]. The crystal packing is stabilized by non-classical hydrogen-bonding contacts between the central sulfur atom (S2) of the S_3_ fragment as acceptor and a C—H of one cyclo­hexyl moiety (C6—H6*B*) as donor, pointing roughly into opposite directions and protruding along the *c*-axis direction (C6—H6*B*⋯S2^i^ and S2⋯H6*B* ^ii^—C6^ii^; symmetry codes: (i) *x*, 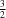 − *y*, 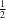 + *z*, (ii): *x*, 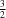 − *y*, −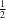 + *z*) (Fig. 3 ▸ and Table 1 ▸).

## Synthesis and crystallization   

The title compound was synthesized based on a modified literature procedure (Magnusson, 1959 ▸). A 20% aqueous solution of ammonium polysulfide (63.9 ml, 187 mmol) was cooled to 273 K and added dropwise over 10 min to stirred cyclo­hexa­none (25.8 ml, 250 mmol) cooled to the same temperature, leading to a uniform mixture of yellow color. Deviating from the reported procedure, addition of colloidal sulfur (4.0 g, 125 mmol), albeit quickly dissolving, leads to liquid–liquid phase separation and a change of color from yellow to green. After stirring for 24 h at 295 K, 100 ml of 10% aqueous acetic acid was added to the reaction mix, which then was extracted in 3 × 50 ml of diethyl ether. The organic fractions were combined and washed with aqueous, saturated NaHCO_3_ (1×100 ml) and water (1×100 ml), before being dried over Na_2_SO_4_. The solvent was reduced to 5 ml *in vacuo* and adsorbed onto isolute® HM-N, prior to purification by flash chromatography (silica 60, 20-45 µm particle diameter, 5 cm column diameter, 50 cm column length, 15 ml min^−1^ ethyl acetate (0–25%) in *n*-hexane, detection by thin layer chromatography and fluorescence quenching at 254 nm). Recrystallization from 0.1 ml mg^−1^ methanol yielded colorless block-like crystals, the identity of which was confirmed by melting point determination (356.5 K). As a result of the lipophilic nature of the analyte, the purity and stability of the colorless product was accessible to supercritical fluid chromatography (stationary phase: Torus DIOL column, mobile phase: scCO_2_ (*A*) and methanol containing 20 mM ammonium formate (*B*), isocratic mode (5% *B*), oven temperature: 313 K). Yield: 5.0 g (14%).

^1^H NMR (400MHz, CDCl_3_) δ 1.45 ppm (*q*, 4H), 1.6 ppm (*m*, 8H), 1.9 ppm (*t*, 8H). ^13^C{^1^H} NMR (101MHz, CDCl_3_) δ 25.4 ppm, 37.8 ppm. IR (FT–IR): (υ cm^−1^) = 2926 (*s*), 1439 (*s*). Elemental analysis calculated for C_12_H_20_S_5_: C 44.40; H 6.21; S 49.39. Found: C 44.72; H 6.03; S 49.25.

## Refinement   

Crystal data, data collection and structure refinement details are summarized in Table 2 ▸. All C-bound hydrogen atoms constitute methyl­ene protons, which were attached in calculated positions (C—H = 0.99 Å) and treated as riding with *U*_iso_(H) = 1.2*U*_eq_(C).

## Supplementary Material

Crystal structure: contains datablock(s) I. DOI: 10.1107/S2056989019007138/fy2136sup1.cif

Structure factors: contains datablock(s) I. DOI: 10.1107/S2056989019007138/fy2136Isup2.hkl

Supporting information file. DOI: 10.1107/S2056989019007138/fy2136Isup3.cml

CCDC reference: 1916548

Additional supporting information:  crystallographic information; 3D view; checkCIF report

## Figures and Tables

**Table 1:** Hydrogen-bond geometry (Å, °)

*D*—H⋯*A*	*D*—H	H⋯*A*	*D*⋯*A*	*D*—H⋯*A*
C6—H6*B*⋯S2^i^	0.99	2.96	3.787 (2)	142

Symmetry code: (i) 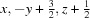.

**Table 2:** Experimental details

Crystal data	
Chemical formula	C_12_H_20_S_5_
*M*_r_	324.58
Crystal system, space group	Monoclinic, *P*2_1_/*c*
Temperature (K)	170
*a*, *b*, *c* (Å)	9.4174 (19), 9.970 (2), 15.877 (3)
β (°)	98.94 (3)
*V* (Å^3^)	1472.6 (5)
*Z*	4
Radiation type	Mo *K*α
μ (mm^−1^)	0.76
Crystal size (mm)	0.39 × 0.36 × 0.28

Data collection	
Diffractometer	Stoe IPDS2T
No. of measured, independent and observed [*I* > 2σ(*I*)] reflections	16257, 4048, 3534
*R*_int_	0.039
(sin θ/λ)_max_ (Å^−1^)	0.691

Refinement	
*R*[*F* ^2^ > 2σ(*F* ^2^)], *wR*(*F* ^2^), *S*	0.028, 0.071, 1.07
No. of reflections	4048
No. of parameters	154
H-atom treatment	H-atom parameters constrained
Δρ_max_, Δρ_min_ (e Å^−3^)	0.35, −0.39

Computer programs: *X-AREA* (Stoe & Cie, 2010 ▸), *SHELXT2018* (Sheldrick, 2015*a* ▸), *SHELXL2018* (Sheldrick, 2015*b* ▸), *XP* in *SHELXTL* (Sheldrick, 2008 ▸), *Mercury* (Macrae *et al.*, 2006 ▸) and *CIFTAB* (Sheldrick, 2015 ▸).

## supplementary crystallographic information

### Crystal data

**Table:**

C_12_H_20_S_5_	*F*(000) = 688
*M**_r_* = 324.58	*D*_x_ = 1.464 Mg m^−^^3^
Monoclinic, *P*2_1_/*c*	Mo *K*α radiation, λ = 0.71073 Å
*a* = 9.4174 (19) Å	Cell parameters from 16821 reflections
*b* = 9.970 (2) Å	θ = 6.3–58.9°
*c* = 15.877 (3) Å	µ = 0.76 mm^−^^1^
β = 98.94 (3)°	*T* = 170 K
*V* = 1472.6 (5) Å^3^	Block, colourless
*Z* = 4	0.39 × 0.36 × 0.28 mm

### Data collection

**Table:**

Stoe IPDS2T diffractometer	3534 reflections with *I* > 2σ(*I*)
Radiation source: fine-focus sealed tube	*R*_int_ = 0.039
Detector resolution: 6.67 pixels mm^-1^	θ_max_ = 29.4°, θ_min_ = 3.1°
ω scans	*h* = −13→12
16257 measured reflections	*k* = −13→13
4048 independent reflections	*l* = −21→21

### Refinement

**Table:**

Refinement on *F*^2^	Primary atom site location: dual
Least-squares matrix: full	Secondary atom site location: difference Fourier map
*R*[*F*^2^ > 2σ(*F*^2^)] = 0.028	Hydrogen site location: inferred from neighbouring sites
*wR*(*F*^2^) = 0.071	H-atom parameters constrained
*S* = 1.07	*w* = 1/[σ^2^(*F*_o_^2^) + (0.035*P*)^2^ + 0.4842*P*] where *P* = (*F*_o_^2^ + 2*F*_c_^2^)/3
4048 reflections	(Δ/σ)_max_ = 0.001
154 parameters	Δρ_max_ = 0.35 e Å^−^^3^
0 restraints	Δρ_min_ = −0.39 e Å^−^^3^

### Special details

**Table:**

Geometry. All esds (except the esd in the dihedral angle between two l.s. planes) are estimated using the full covariance matrix. The cell esds are taken into account individually in the estimation of esds in distances, angles and torsion angles; correlations between esds in cell parameters are only used when they are defined by crystal symmetry. An approximate (isotropic) treatment of cell esds is used for estimating esds involving l.s. planes.

### Fractional atomic coordinates and isotropic or equivalent isotropic displacement parameters (Å^2^)

**Table:**

	*x*	*y*	*z*	*U*_iso_*/*U*_eq_	
S1	0.31425 (3)	0.61591 (3)	0.37879 (2)	0.02374 (8)	
S2	0.38015 (3)	0.68085 (4)	0.26964 (2)	0.02454 (8)	
S3	0.57525 (3)	0.59559 (3)	0.26792 (2)	0.02661 (8)	
S4	0.65189 (4)	0.83978 (3)	0.38610 (2)	0.02869 (9)	
S5	0.58752 (3)	0.72925 (4)	0.48039 (2)	0.02763 (8)	
C1	0.38995 (13)	0.73599 (13)	0.46088 (8)	0.0209 (2)	
C2	0.33611 (14)	0.87799 (13)	0.44215 (9)	0.0248 (3)	
H2A	0.357462	0.905539	0.385535	0.030*	
H2B	0.388180	0.939484	0.485236	0.030*	
C3	0.17466 (15)	0.89094 (14)	0.44305 (10)	0.0285 (3)	
H3A	0.146241	0.986281	0.435293	0.034*	
H3B	0.121809	0.839446	0.394775	0.034*	
C4	0.13306 (16)	0.83957 (14)	0.52607 (9)	0.0295 (3)	
H4A	0.175028	0.899044	0.573399	0.035*	
H4B	0.027179	0.842519	0.522274	0.035*	
C5	0.18500 (16)	0.69697 (15)	0.54533 (9)	0.0298 (3)	
H5A	0.132692	0.635465	0.502332	0.036*	
H5B	0.163361	0.670101	0.602005	0.036*	
C6	0.34657 (16)	0.68404 (15)	0.54448 (8)	0.0280 (3)	
H6A	0.374816	0.588653	0.552229	0.034*	
H6B	0.399212	0.735309	0.592926	0.034*	
C7	0.71249 (13)	0.72138 (13)	0.31199 (8)	0.0219 (2)	
C8	0.84149 (14)	0.64097 (14)	0.35539 (8)	0.0252 (3)	
H8A	0.912118	0.703127	0.387439	0.030*	
H8B	0.809187	0.578098	0.396805	0.030*	
C9	0.91472 (15)	0.56165 (15)	0.29197 (9)	0.0289 (3)	
H9A	1.000340	0.515105	0.322612	0.035*	
H9B	0.847733	0.492960	0.263730	0.035*	
C10	0.95977 (15)	0.65513 (16)	0.22523 (9)	0.0291 (3)	
H10A	1.004584	0.602264	0.183578	0.035*	
H10B	1.032100	0.719631	0.253151	0.035*	
C11	0.83096 (15)	0.73143 (15)	0.17883 (9)	0.0280 (3)	
H11A	0.762969	0.667255	0.146541	0.034*	
H11B	0.863656	0.794214	0.137496	0.034*	
C12	0.75366 (15)	0.81008 (14)	0.24074 (9)	0.0264 (3)	
H12A	0.665632	0.851094	0.208864	0.032*	
H12B	0.816840	0.883488	0.266381	0.032*	

### Atomic displacement parameters (Å^2^)

**Table:**

	*U*^11^	*U*^22^	*U*^33^	*U*^12^	*U*^13^	*U*^23^
S1	0.02576 (15)	0.02375 (16)	0.02281 (15)	−0.00552 (12)	0.00722 (11)	−0.00510 (12)
S2	0.02277 (15)	0.03175 (17)	0.01882 (15)	0.00001 (12)	0.00232 (11)	−0.00116 (12)
S3	0.02379 (15)	0.02383 (16)	0.03371 (18)	−0.00428 (12)	0.00918 (13)	−0.00932 (12)
S4	0.02747 (16)	0.02263 (16)	0.03820 (19)	−0.00624 (13)	0.01207 (14)	−0.01101 (13)
S5	0.02292 (15)	0.0385 (2)	0.02067 (15)	0.00173 (13)	0.00072 (11)	−0.00585 (13)
C1	0.0228 (5)	0.0217 (6)	0.0186 (5)	−0.0003 (5)	0.0042 (4)	−0.0028 (4)
C2	0.0276 (6)	0.0192 (6)	0.0296 (7)	−0.0005 (5)	0.0106 (5)	−0.0007 (5)
C3	0.0284 (6)	0.0247 (7)	0.0349 (7)	0.0046 (5)	0.0122 (5)	0.0043 (5)
C4	0.0309 (7)	0.0280 (7)	0.0327 (7)	0.0002 (5)	0.0146 (6)	−0.0021 (6)
C5	0.0361 (7)	0.0289 (7)	0.0274 (7)	−0.0022 (6)	0.0145 (5)	0.0026 (5)
C6	0.0350 (7)	0.0306 (7)	0.0195 (6)	0.0034 (6)	0.0075 (5)	0.0033 (5)
C7	0.0211 (5)	0.0203 (6)	0.0247 (6)	−0.0023 (5)	0.0049 (4)	−0.0037 (5)
C8	0.0245 (6)	0.0279 (6)	0.0233 (6)	0.0015 (5)	0.0040 (5)	0.0019 (5)
C9	0.0265 (6)	0.0309 (7)	0.0299 (7)	0.0071 (5)	0.0065 (5)	0.0015 (5)
C10	0.0236 (6)	0.0381 (8)	0.0268 (7)	−0.0002 (6)	0.0073 (5)	−0.0016 (6)
C11	0.0283 (6)	0.0333 (7)	0.0230 (6)	−0.0023 (6)	0.0059 (5)	0.0033 (5)
C12	0.0276 (6)	0.0216 (6)	0.0304 (7)	−0.0009 (5)	0.0059 (5)	0.0032 (5)

### Geometric parameters (Å, º)

**Table:**

S1—C1	1.8306 (13)	C5—H5B	0.9900
S1—S2	2.0353 (6)	C6—H6A	0.9900
S2—S3	2.0284 (6)	C6—H6B	0.9900
S3—C7	1.8579 (13)	C7—C8	1.5274 (18)
S4—C7	1.8200 (13)	C7—C12	1.5325 (18)
S4—S5	2.0261 (6)	C8—C9	1.5267 (19)
S5—C1	1.8393 (13)	C8—H8A	0.9900
C1—C2	1.5175 (18)	C8—H8B	0.9900
C1—C6	1.5382 (18)	C9—C10	1.520 (2)
C2—C3	1.5282 (19)	C9—H9A	0.9900
C2—H2A	0.9900	C9—H9B	0.9900
C2—H2B	0.9900	C10—C11	1.522 (2)
C3—C4	1.5211 (19)	C10—H10A	0.9900
C3—H3A	0.9900	C10—H10B	0.9900
C3—H3B	0.9900	C11—C12	1.5277 (19)
C4—C5	1.519 (2)	C11—H11A	0.9900
C4—H4A	0.9900	C11—H11B	0.9900
C4—H4B	0.9900	C12—H12A	0.9900
C5—C6	1.529 (2)	C12—H12B	0.9900
C5—H5A	0.9900		
			
C1—S1—S2	105.16 (5)	C1—C6—H6B	109.2
S3—S2—S1	105.95 (3)	H6A—C6—H6B	107.9
C7—S3—S2	106.89 (5)	C8—C7—C12	111.17 (10)
C7—S4—S5	106.55 (5)	C8—C7—S4	110.87 (9)
C1—S5—S4	105.47 (5)	C12—C7—S4	104.10 (9)
C2—C1—C6	110.96 (10)	C8—C7—S3	105.87 (9)
C2—C1—S1	112.92 (9)	C12—C7—S3	110.64 (9)
C6—C1—S1	105.53 (9)	S4—C7—S3	114.31 (7)
C2—C1—S5	111.44 (9)	C9—C8—C7	112.56 (11)
C6—C1—S5	103.87 (9)	C9—C8—H8A	109.1
S1—C1—S5	111.58 (7)	C7—C8—H8A	109.1
C1—C2—C3	112.31 (11)	C9—C8—H8B	109.1
C1—C2—H2A	109.1	C7—C8—H8B	109.1
C3—C2—H2A	109.1	H8A—C8—H8B	107.8
C1—C2—H2B	109.1	C10—C9—C8	110.22 (12)
C3—C2—H2B	109.1	C10—C9—H9A	109.6
H2A—C2—H2B	107.9	C8—C9—H9A	109.6
C4—C3—C2	111.73 (12)	C10—C9—H9B	109.6
C4—C3—H3A	109.3	C8—C9—H9B	109.6
C2—C3—H3A	109.3	H9A—C9—H9B	108.1
C4—C3—H3B	109.3	C9—C10—C11	110.86 (11)
C2—C3—H3B	109.3	C9—C10—H10A	109.5
H3A—C3—H3B	107.9	C11—C10—H10A	109.5
C5—C4—C3	111.80 (11)	C9—C10—H10B	109.5
C5—C4—H4A	109.3	C11—C10—H10B	109.5
C3—C4—H4A	109.3	H10A—C10—H10B	108.1
C5—C4—H4B	109.3	C10—C11—C12	111.65 (12)
C3—C4—H4B	109.3	C10—C11—H11A	109.3
H4A—C4—H4B	107.9	C12—C11—H11A	109.3
C4—C5—C6	111.52 (12)	C10—C11—H11B	109.3
C4—C5—H5A	109.3	C12—C11—H11B	109.3
C6—C5—H5A	109.3	H11A—C11—H11B	108.0
C4—C5—H5B	109.3	C11—C12—C7	112.29 (11)
C6—C5—H5B	109.3	C11—C12—H12A	109.1
H5A—C5—H5B	108.0	C7—C12—H12A	109.1
C5—C6—C1	112.17 (11)	C11—C12—H12B	109.1
C5—C6—H6A	109.2	C7—C12—H12B	109.1
C1—C6—H6A	109.2	H12A—C12—H12B	107.9
C5—C6—H6B	109.2		

### Hydrogen-bond geometry (Å, º)

**Table:**

*D*—H···*A*	*D*—H	H···*A*	*D*···*A*	*D*—H···*A*
C6—H6*B*···S2^i^	0.99	2.96	3.787 (2)	142

Symmetry code: (i) *x*, −*y*+3/2, *z*+1/2.
